# Significant Comparative Characteristics between Orphan and Nonorphan Genes in the Rice (*Oryza sativa L.*) Genome

**DOI:** 10.1155/2007/21676

**Published:** 2007-09-18

**Authors:** Wen-Jiu Guo, Ping Li, Jun Ling, Shao-Ping Ye

**Affiliations:** ^1^Department of Biology, An-Kang University, An-kang 725000, Shaan-Xi, China; ^2^Rice Research Institute, Si-Chuan Agricultural University, Wen-Jiang, Cheng-Du 611130, Si-Chuan, China

## Abstract

Microsatellites are short tandem repeats of one to six bases in genomic DNA. As microsatellites are highly polymorphic and play a vital role in gene function and recombination, they are an attractive subject for research in evolution and in the genetics and breeding of animals and plants. Orphan genes have no known homologs in existing databases. Using bioinformatic computation and statistical analysis, we identified 19,26 orphan genes in the rice (*Oryza sativa ssp. Japanica cv. Nipponbare*) proteome. We found that a larger proportion of orphan genes are expressed after sexual maturation and under environmental pressure than nonorphan genes. Orphan genes generally have shorter protein lengths and intron size, and are faster evolving. Additionally, orphan genes have fewer PROSITE patterns with larger pattern sizes than those in nonorphan genes. The average microsatellite content and the percentage of trinucleotide repeats in orphan genes are also significantly higher than in nonorphan genes. Microsatellites are found less often in PROSITE patterns in orphan genes. Taken together, these orphan gene characteristics suggest that microsatellites play an important role in orphan gene evolution and expression.

## 1. INTRODUCTION

Microsatellites, also known as short tandem repeats (STRs) or simple sequence repeats (SSRs), are tandem repeats of 1–6 base pairs in genomic sequences [1]. As microsatellites are highly polymorphic [2], they are useful in DNA genotyping [3], fingerprinting [4], and forensic science [5– 7]. Microsatellites also have intrinsic functions in gene expression and regulation and in chromosomal recombination [1]. Additionally, microsatellites have strong evolutionary ties to genes and genomes [8]. As genome diversity is nonrandom, well structured, and correlates with stress and higher environmental heterogeneity [9], excess microsatellite loci play significant roles not only in genome stability but also in genomic characteristics such as codon bias evolution [10]. Orphan genes in a genome are these that have no known match when aligned in the current database, for example, NBCI nr, at some BLAST e-cutoff. In this paper, we studied the common features of highly informative and polymorphic microsatellites and orphan genes in the rice genome.

In *Drosophila melanogaster*, the characteristics of orphan genes are well documented [11]. Compared with nonorphan genes, orphan genes are shorter, evolve more rapidly, and are expressed at a higher percentage in the adult stage. Understanding these features is useful in rice genetics and breeding, if it is true that orphan genes are expressed at a higher percentage in adult stage and may govern economic traits such as grain size, weight, and others related to grain yield or quality. These genes may be selection targets for breeders while selection bases on gene in DNA level. The research on orphan genes not only implicates meaningful evolutionary fundamentals of organisms but also can assist plant and animal breeding. The goal of this paper is not to decipher the function of individual orphan genes in detail but to obtain the hallmark of orphan genes in the features of microsatellite content, constitution, variability and expression difference of genes in comparison with nonorphan genes.

The main routes to new gene formation include exon shuffling, gene duplication, retroposition, mobile elements, lateral gene transfer, gene fusion fission, and *de novo* origination [12]. Although no one has yet described the prerequisites for gene origination from these events and we cannot give such knowledge, the work in this study supplied many patterns by comparison of microsatellite, variability and expression between orphan and nonorphan genes to help to understand the knowledge. The characteristics of orphan genes can be used to infer the prerequisites for the new gene evolution.

## 2. MATERIALS AND METHODS

### 2.1. Data collection

We used the IRSGP (international rice sequencing genome project) rice (*Oryza sativa L. ssp. Japonica cv. Nipponbare*) whole genomic sequences and annotation release 2 by TIGR (The Institute for Genomic Research) [13] as the sequence and EST-evidenced annotation dataset. The full-length cDNA dataset [14] was used to determine the expression stages of orphan and nonorphan genes within the rice life cycle. The PROSITE database [15] and ps scan for win32 [16] were applied.

### 2.2. Computation and analysis

We applied the Perl script written by Temnykh et al. to compute microsatellites in DNA sequences [17]. The microsatellite length criterion of 1–5 base motifs perfectly tandemly repeated at least three times and of a total size of at least 12 bases has been used by others [18, 19]. Thus a mononucleotide motif must have been repeated 12 times, a dinucleotide motif repeated 6 times, a trinucleotide motif repeated 4 times, and tetra- or pentanucleotide motifs repeated 3 times in order to be considered a microsatellite. BioPerl tools were used for various computations and analyses [20]. Interactions between microsatellites and PROSITE patterns were conducted by computing microsatellite loci in CDS and PROSITE patterns by transforming the coordinates of the computed PROSITE patterns into DNA coordinates with base 1 DNA *start position = protein start* *3− 2 and end position = protein end *3, and then finding the intersections.

### 2.3. Definition of microsatellite content and statistical methods used

We defined the microsatellite content as total microsatellites within a moving window divided by the window size, and then multiplied by a constant 1 × 10^6^. The unit for microsatellite content is microsatellite bases/megabases of genomic sequence. The definition we proposed represents the true content of microsatellites in a genomic sequence.

We made the microsatellite content in an individual sequence a statistical observation and all the observations were divided into two groups of orphan and nonorphan genes. We employed nonparameter statistics to test the differences for the comparisons.

## 3. RESULTS

### 3.1. Orphan genes in the rice genome

**Figure 1:**
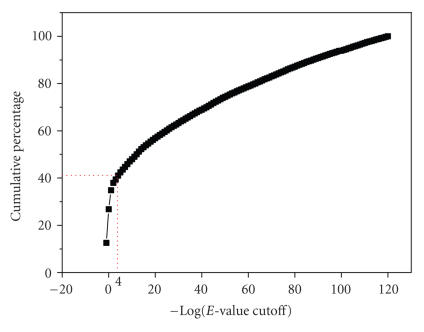
The cumulative percentages of orphan genes against different BLAST E-cutoffs. The x-axis indicates the BLAST E-cutoffs following negative logarithmic transformation, and the y-axis indicates the number of orphan genes obtained at different E values. The curve rises sharply as E values drop and then levels off. The turning point is around the E-cutoff = 10^−4^. At E-cutoff = 10^−4^, we obtained 18,398 orphans out of a total of 59,712, which accounts for 30.8% of the total protein sequences of the annotated proteome.

The rice annotated proteome, which has 59,712 protein sequences, was aligned to the NCBI nr database using the NCBI BLAST program suite [21, 22]. We used 10^−4^ as the expectation (E)-cutoff for defining the number of orphan genes. A similar E-cutoff has previously been applied to the *Drosophila* genome [23]. We compared the number of orphan genes to that of the E-cutoff. Figure 1 shows that, at a BLAST E-cutoff of 10^−4^, the cumulative percentage of orphan genes reached 41%. Beyond that E-cutoff, the cumulative percentage increased slowly. In comparison, at an E-cutoff of 10^−3^ to 10^−6^, the cumulative percentage of orphan genes in *Drosophila* is 26–29% [11]. This indicated that most orphan genes are species specific and not related to the number of genes detected in other species.

To correct errors in annotation of the rice genome, we used the *all. TU_model.brief_info* file in the TIGR annotation dataset to validate the orphan gene number with EST matches. We obtained 1,926 orphan protein sequences, which is a sharp decrease compared to 18,398 of total orphan genes in BLAST result; it is possible that a large proportion of orphan genes in the initial rice genome annotation are artifacts [24–26]. Therefore, the most reliable orphan gene calls should be made from matches in the EST dataset. All the orphan genes we identified had such EST matches (see the Supplementary Table available online at doi:10.1155/2007/21676).

### 3.2. Comparison of expression rates between orphan and nonorphan genes in different tissues and after injury or hormone treatment

**Figure 2:**
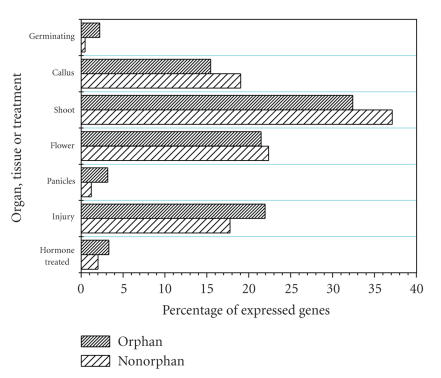
Comparison of the number of orphan and nonorphan genes expressed in different tissues or following injury or hormone treatment. Germinating: shoot and roots of germinating seeds in the library; callus: callus library; shoot: green shoot, shoot, shoot and callus, shoot and root of germinating seeds and mixed shoot (normalized library); flower: flower library; panicles: mixture of library 21 and library 22 (panicles less than 5 cm stage and panicles two weeks after flowering), mixture of library 29 and library 33 (panicles mixture of one, two, and three weeks after flowering and supermix), mixture of library 29 and library 35 (panicles mixture of one, two, and three weeks after flowering), mixture of library 30 and library 34 (panicles mixture of one, two, and three weeks after flowering and supermix), mixture of library 30 and library 36 (panicles mixture of one, two, and three weeks after flowering), and mixture of library 19 and library 20 (panicles more than 5 cm stage and panicles one day after flowering); injury: Cd-treated callus, cold-treated callus, etiolated shoot, heat-treated callus, and UVC irradiated shoot; hormone treated: ABA (abscisic acid) ABA-treated callus and NAA (naphthaleneacetic acid)-treated callus. All the count values (the values shown are the corresponding percentages) were tested by Pearson Chi-Square by means of whole and separate data pairs (for example, germinating and callus can form a data pair). The whole table test is significant, *P* = 1.4 × 10^−46^.

We used ready made full-length cDNA libraries from different tissues and treatments [14]. The dataset contains 32,127 full-length cDNAs (release date June 17, 2004) and can be accessed at ftp://cdna01.dna.affrc.go.jp/pub/data/20040617. We could only map 25,204 of these sequences on TIGR's pseudomolecules of Release 2. There are 35 libraries in the dataset. We summed the 35 libraries into five stages of development and two different treatments to clearly compare the difference of expression under normal and environmental presses on a larger scale. We counted the number of orphan and nonorphan genes previously defined in the method section, and then compute the percentage of each group by dividing by the total number of the whole. Figure 2 shows the result.

In the early stages of rice growth, including the callus, shoot, and flower, a large proportion of total genes in the genome is expressed, and the percentage of nonorphan genes expressed is significantly higher than that of orphan genes. In the late stage (panicles) the situation was reversed, with orphan genes expressed at a higher percentage than nonorphan genes. These phenomena are quite similar to those seen in *Drosophila* [11]. Figure 2 also shows that the flowering stage constitutes the boundary of dynamic equilibrium of expression rates between orphan and nonorphan genes. This suggests that orphan gene expression dominates only after sexual maturation, except when additional stresses are applied (see the following). The germinating stage may either be the exception to the trend or an example of expression of orphan genes due to environmental stresses.

Injured or hormone-treated tissues expressed a large proportion of orphan genes, possibly indicating that a majority of orphan genes are nonessential and are responsible for responding to environmental stresses. Injury or hormone treatment caused excess expression of orphan genes, which may be indicative of the flexibility of the genome and gene expression, with orphan genes being the most flexible.

### 3.3. Intron length and mismatch rates of orphan genes

A previous study showed that, in *Drosophila*, orphan or nascent genes are shorter than nonorphan genes [11]. To test whether the same is true in rice, we conducted a similar analysis. Table 1 shows that protein length in nonorphan genes is significantly longer than orphan genes. This corresponds to the situation in *Drosophila* and other species and may be a common characteristic of orphan genes. The average intron size in orphan genes is also significantly shorter than in nonorphan genes (see Table 1). This indicates that the orphan gene may be a nascent gene that is fast evolving, as genes in prokaryotes also have fewer or no introns. Larger introns occur preferentially in regions of low recombination which are generally conserved and have a deleterious effect [27]. Since orphan genes have characteristics of shorter introns, they show nonconservative features in gene evolution.

Nascent or orphan genes are generally fast evolving in *Drosophila* and other species [11, 12, 28]. To test whether this is also true in rice, we downloaded the indica EST database from the TIGR FTP site (ftp://ftp.tigr.org/ pub/data/Eukaryotic_Projects/o_sativa/annotation_dbs/EST) and aligned the orphan and nonorphan genes used in this study to the indica EST with an E-cutoff equivalent to 10^−20^. We then calculated the mismatch rate in each HSP (high scoring pair). The mismatch rates in the panicles and callus tissues between orphan and nonorphan genes were compared (see Table 2). In either panicles or callus, the average mismatch rate in orphan genes was significantly higher than that in nonorphan genes. These results suggest that orphan genes as a whole are the faster evolving component of the rice genome, a finding that is novel in rice. The phenomena observed in this study are similar to those described in studies in *Drosophila* using different methods. Therefore, it may be a common rule that orphan genes are always shorter and evolve more rapidly. This is also consistent withthe negative relationship between protein length and a gene's evolutionary rate [29].

### 3.4. Microsatellite comparison between orphan and nonorphan genes

There are more than eight pathways leading to the origin of orphan genes [11, 12], but no one has been able to address what the prerequisite for survival of orphan genes is or why orphan genes are faster evolving and not eliminated by natural selection. Because microsatellites mutate quickly and the mutation takes the form of polymorphic lengths, and the mismatch rate and intron length of orphan genes are significantly different from nonorphan genes, it seems reasonable to associate the fast mutation rate of orphan genes with microsatellites.

**Figure 3:**
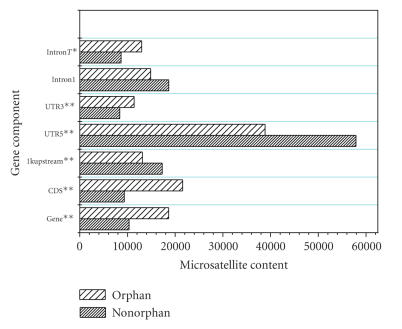
Microsatellite content among gene components. IntronT and intron1 represent the microsatellite content in all introns and in the first intron of a gene, respectively. CDS represents the microsatellite content in CDS (coding sequence). In intronT and CDS, the microsatellite content of orphan genes is significantly higher than nonorphan genes. All the data pairs are highly significant (*P* ≪ .01) in the Mann-Whitney U test. ** Probability <.05; *probability <.01.

To assess this possibility, we analyzed orphan and nonorphan genes with regard to the microsatellite content in different gene elements (see Figure 3). The most remarkable feature is that the microsatellite content of orphan genes in CDS (coding sequence) is extremely high. As a microsatellite mutation is generally a length polymorphism (i.e., a change of repeat number), this result indicated that microsatellites act as mutation reservoirs in orphan genes more effectively than in nonorphan genes, reflecting the higher mutation rate of orphan genes. The reservoir is not only in CDS but also in other components, including the intron, untranslated regions (UTRs), and the promoter and enhancer. Higher microsatellite content in CDS and introns coincides with higher microsatellite content in the whole gene and appears to be the major contributor to the high microsatellite content of the gene. The UTRs play vital roles in transcription: the 5′UTR influences the transcriptional start position and is tissue specific [30], and a faux 3′UTR promotes aberrant termination [31]. Figure 3 contrasts the difference in microsatellite content between the 5′UTR and the 3′UTR. Microsatellite content in the 5′UTR is extremely high, particularly in nonorphan genes, suggesting that the 5′UTR relates to the major variable region of gene, because higher microsatellite content may have more chance to mutate. The differences may be due to some requirement(s) of gene evolution that remains unexplained.

**Figure 4:**
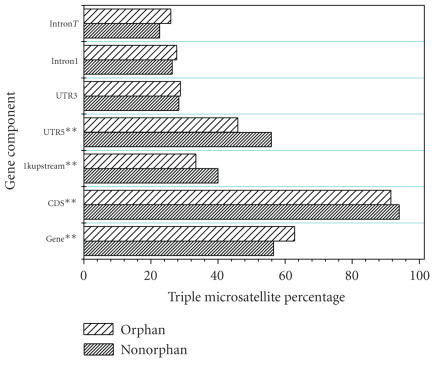
Triplet microsatellite content as a percentage of the total mononucleotide to pentanucleotide microsatellite content of orphan and nonorphan genes. IntronT and intron1 represent microsatellite content in all introns and in the first intron of a gene, respectively. All the data pairs were tested by the Mann-Whitney U test. ** Probability <.05; *probability <.01.

Figure 4 shows the triple (trinucleotide) microsatellite percentage of each gene component. The triple microsatellite percentages in the entire gene, the first intron, and all the introns combined were higher in orphan genes. While the absolute values of the triple microsatellite percentages in the CDS of both orphan and nonorphan genes were very high, the percentages were not significantly different. We speculate that triple microsatellites may be scattered among other gene components (mainly the introns and CDS) rather than located only in the CDS. The introns are identical to the CDS in terms of microsatellite content. Figure 4 also demonstrates that the quantity of microsatellites rather than motif frequency contributes to microsatellite content.

### 3.5. Comparison of associations between microsatellite and PROSITE patterns in orphan and nonorphan genes

PROSITE is a database of biologically meaningful motifs and patterns of proteins [15]. We examined the number of PROSITE patterns that overlapped with microsatellite arrays in the CDS of orphan and nonorphan genes. We used the ScanProsite program [16] to scan PROSITE patterns in orphan and nonorphan genes and collected the coordinates of each pattern in a sequence into a relational database table. The microsatellite scanning program also provided the coordinates of each microsatellite locus in a sequence and then converted the PROSITE pattern coordinates into DNA coordinates so that the relationship between microsatellites and PROSITE patterns could be obtained. To compare the flexibility between orphan and nonorphan genes, we counted the number of PROSITE patterns, calculated the average size of each PROSITE pattern, and determined the number of microsatellite loci that overlapped the PROSITE patterns in the CDS of each gene. PROSITE patterns include duplicate ones in a sequence for the purpose of counting numbers of such patterns in a sequence and comparing complexity of protein between orphan and nonorphan genes.

First, we compared the protein complexity between orphan and nonorphan genes in terms of PROSITE pattern statistics by comparing the number of PROSITE patterns and the average pattern sizes using the Mann-Whitney test. As seen in Table 3, the number of patterns in nonorphan genes is significantly higher than in orphan genes. However, the average PROSITE pattern size of orphan genes is larger than nonorphan genes. These results indicate that nonorphan genes are more complex than orphan genes, whereas orphan genes have greater PROSITE pattern size. Thus, nonorphan genes have a lower capacity for mutations than orphan genes, and orphan genes are more flexible than nonorphan genes in terms of protein complexity. These results also support the aforementioned conclusion that orphan genes are generally fast evolving.

Second, we compared the associations between microsatellites and PROSITE patterns. We calculated three indicators of such associations: number of microsatellite loci outside PROSITE patterns, number of microsatellites overlapping PROSITE patterns, and number of microsatellites contained within PROSITE patterns of orphan and nonorphan genes. Using the Chi-Square test of crosstab, we found that this association is statistically highly significant, with the significant probability of Pearson Chi-Square equal to 1.36744×10^−20^. Table 4 showed that a large proportion of microsatellites in nonorphan genes are found within PROSITE patterns (97.1%), indicating that microsatellite mutations in nonorphan genes are more likely to involve PROSITE patterns, so that the mutation pressure is higher. In orphan genes the situation is reversed in that the microsatellites are more likely to be found outside protein patterns. Microsatellites outside protein patterns may be crucial for gene evolution. A classic example is the AFGP-protease gene (AFGP: ice-binding antifreeze glycoprotein) where, in the evolutionary process of the gene, a (gt)n microsatellite found before the repeats of Thr-Ala-Ala-coding element presumably facilitated duplication of the ancestral Thr-Ala-Ala-coding element through replication slippage or gene conversion [28, 32]. In most cases, microsatellite polymorphism causes quantitative change of gene expression for a trait rather than lethal mutation and does not reduce fitness of selection [33]. In the presence of microsatellites, gene evolution is facilitated, and the results of the mutations more easily survive. Thus, our evidence shows that microsatellites may be a necessary prerequisite for the origin of new genes.

## 4. DISCUSSION

Orphan genes may have at least two levels of divergence in gene evolution. The first level is expression rate. We found that when rice plants were exposed to environmental stresses, either injury or hormone treatment, a larger proportion of orphan genes were expressed. Two models to explain how new genes evolve, the waiting model and the immediate model, have been proposed by Long et al. [12]. Our results support the waiting model. In this model, genes can be divided into two types, essential and nonessential, in terms of conservation in function. The essential genes are stably expressed according to a programmed time series. Nonessential genes are only expressed in specific tissues, thereby programming tissue function or imparting species-specific and species-phenotype-specific attributes and/or responses to environmental stress. All the nonessential genes have already been established during evolution, but their expression is not predictable or behaves with nonintegrality in that not all genes are expressed in a presumed time series but rather in specific tissues or under environmental stress. When these genes are not expressed, they exhibit recessivity and their presence in the genome does not reduce fitness [34]. It may be that only genes having higher microsatellite content have this property. Microsatellite length polymorphism in genes leads to protein diversity due to the generation of alternate genes with overlapping or redundant function. For this reason, genome diversity is nonrandom and correlates with stress and environmental heterogeneity [9].

The second level of orphan gene divergence is mutation rate. As demonstrated in this and other studies [11, 12], orphan genes are generally fast evolving. Higher microsatellite content in the CDS, introns, and UTRs (see Figure 3), in addition to the characteristics of length polymorphism of microsatellites, provide the second reservoir for gene evolution. From the evidence of the evolution of the AFGP-protease gene [12, 32], microsatellites not only help generate mutations but also facilitate gene evolution.

Abundant microsatellites are a major source of mutations that have a quantitative effect on phenotype but do not reduce fitness, broadly implicating the molecular processes of evolutionary adaptation, including the evolutionary control of the mutation process [33]. These mutations aid the survival and spatiotemporal adaptations of the organism under constantly fluctuating natural environments [1, 9]. Microsatellites are more common in eukaryotic genomes. The above conclusion supports the new idea in evolutionary biology that nascent genes evolve towards nonessential functions that are quantitative rather than qualitative in expression and gradually become expansive and divergent in phenotype. Such a trend implicates the vitally important buffering role of microsatellites in the progress of eukaryotic evolution. In fact, the buffering means more elaborate modulation of gene expression, and thus more quantitative phenotypes are possible.

## 5. CONCLUSION

Fewer plant genomes have been sequenced compared with animals or microbes. Previous research indicates that genes may originate from either the plant, animal, or microbial kingdoms and cross over to other kingdoms, or at the very least, that rice gene homologs can be found in patterns other than the plant kingdom [14]. Therefore, if a verifiable gene or genomic sequence contains no homologs in the current genomic databases, then the gene should be considered an orphan. Orphan genes are generally species specific, so they are independent of the current database size. The orphan genes identified in this study should be reliable—at least the statistical attributes of orphan genes are always identical. Rice orphan genes generally evolve rapidly and are expressed in the anaphase of plant generation or under environmental pressures. Microsatellites act as efficacious reservoirs of evolution and expression of orphan genes.

In cereal crop breeding, the most economical traits that are selected have been found to develop at later stages,that is, at the same time at which a large proportion of orphan genes are expressed. Artificial selection against orphan genes thus would be more efficient than selection against nonorphan genes; moreover, the microsatellites in orphan genes could be used directly as markers to facilitate breeding programs. Thus, the marker-assisted selection of genes active in the later stages of development may be one economically important application of microsatellites in orphan genes.

## Supplementary Material

The orphan and nonorphan genes are concluded using NCBI-BLAST program as described in the materials and methods section in the paper. The front part of table is the 1926 orphan genes and later is the 20439 nonorphan genes. The gene name column in the table is the TIGR's model name and the description column is TIGR annotation. The sequences of each gene can be used as base sequences to design microsatellite marker primers for genetic analysis and molecular marker assistant breeding. Markers deduced from orphan genes should be more efficient and that from nonorphan genes less efficient according to the inferences in the discussion and conclusion sections in the paper.

## Figures and Tables

**Table 1:** Comparison of average protein length and intron size between orphan and nonorphan genes using ESTs.

	Nonorphan gene	Orphan gene	Probability
Protein length	583	245	0
Intron size	2277.959428	1474.6711	2.6202E-52

The statistical test of Mann-Whitney U was conducted. Intron size is an average of the sum of all the introns within a gene. The table shows that both average protein length and intron size are highly significant.

**Table 2:** Comparison of average mismatch rates in high similarity pairs (HSPs) of *indica-janpanica* EST alignments at an E-cutoff = 10^−20^ in different tissues.

Tissue	Nonorphan gene	Orphan gene	Probability
Panicles	5.8875806	6.066929	0.015195
Callus	5.9784517	6.200713	0.003167

The mismatch rate = 100 − identity rate. The mismatch rate includes indels (insert and deletes) and substitutions in HSP of the BLAST alignment. The mismatch rates in both tissues are statistically significant using the Mann-Whitney U test.

**Table 3:** Comparison of number of PROSITE patterns and average pattern size in CDSs of orphan and nonorphan genes.

Indicators	Nonorphan gene	Orphan gene	Mann-Whitney probability
Number of PROSITE Patterns	33.76025	15.93652	0
Average PROSITE Pattern size	6.825061	6.965661	3.36221E-26

The table shows the PROSITE pattern complexity of orphan and nonorphan genes. The number includes repetitive PROSITE patterns in the sequence. The Mann-Whitney U test was applied.

**Table 4:** Interaction between microsatellite loci and PROSITE patterns in orphan and nonorphan genes.

Interaction	Nonorphan gene	Orphan gene
Microsatellite loci outside PROSITE patterns	482 (2.9%)	118 (6.6%)
Microsatellite loci overlapping PROSITE patterns	0	2 (0.1%)
Microsatellite loci within PROSITE patterns	16345 (97.1%)	1666 (93.3%)

The table was tested by Chi-Square test of crosstab. The significant probability of Pearson Chi-Square was 1.36744 × 10^−20^. The number includes repetitive PROSITE patterns.
